# Molecular Dynamics Study of the Changes in Conformation of Calmodulin with Calcium Binding and/or Target Recognition

**DOI:** 10.1038/s41598-019-47063-1

**Published:** 2019-07-23

**Authors:** Hiroshi Kawasaki, Natsumi Soma, Robert H. Kretsinger

**Affiliations:** 1 Department of Medical Life Science, Graduate School of Medical Life Science, Yokohama City University, Suehiro-cho, Tsurumi-ku, Yokohama, 230-0045 Japan; 2 Department of Biology, University of Virginia, Charlottesville, VA 22904 USA

**Keywords:** Molecular conformation, Intracellular signalling peptides and proteins

## Abstract

Calmodulin is a calcium binding protein with two lobes, N-lobe and C-lobe, which evolved from duplication and fusion of a single precursor lobe of a pair of EF-hand. These two lobes of calmodulin show subtle differences in calcium binding and target recognition; these are important for the functions of calmodulin. Since the structures, especially main chain conformations, of two EF-lobes in holo-form are quite similar; this is a good example to evaluate the effect of side chains for structural dynamics. We analyzed the structure of calmodulin using molecular dynamics and found differences in conformational ensembles between N- and C-lobes. We also showed the mutant structures created by homology modeling could reproduce the difference of dynamic motion between N- and C-lobes.

## Introduction

Calmodulin functions as a major calcium sensor in cells of eukaryotes; it binds Ca^2+^ ions and transmits the calcium signal to its various targets^1^. The conformational changes induced by calcium and/or target binding are crucial to the functions of calmodulin^2^. Calmodulin binds four Ca^2+^ ions via four EF-hands^3,4^. EF-hand is a helix-loop-helix motif about 30 amino acid residues long. A pair of EF-hands makes a structural unit called an EF-lobe. The two EF-lobes of calmodulin show subtle differences in calcium binding and target recognition; these are important for the functions of calmodulin. We made a plot of the conformational landscape of helix positions of calmodulin lobes by alignment with the pseudo-two fold axis of a pair of EF-hands (EF-lobe)^5–7^. There are two lines in the plot, each of which is an inferred path of open/close of the EF-lobe based on the observed conformational continuum of EF-lobes from many EF-hand proteins^5^. This path for open/close of the EF-lobe is interpreted in the context of the structural changes of the EF-lobe^7^. The plot shows a conformational landscape; this is useful in evaluating the effects of mutation on the conformation of the EF-lobe and/or on the stability and change of the EF-lobe by molecular dynamic (MD) simulation. We analyzed the conformational flexibility of EF-lobe and the difference between N-lobe and C-lobe by MD, and evaluated the method to identify the responsible amino acid residues for the difference between two lobes. Two lobes of calmodulin show subtle differences in calcium binding and target recognition; these are important for the functions of calmodulin. Since the structures, especially main chain conformations, of two EF-lobes in holo-form are quite similar; this is a good example to evaluate the effect of side chains for structural dynamics.

## Results and Discussion

Calmodulin is composed of four EF-hands, each two of which forms a lobe (EF-lobe). Two EF-lobes, N-lobe and C-lobe, of calmodulin are structurally almost independent and show large conformational change upon the binding of calcium. We named four EF-hands as EF1, EF2, EF3 and EF4 from N-terminal side. EF-hand is a helix-loop-helix motif. The helix E is a helix incoming from N-terminus to calcium binding loop. The helix F is a helix exiting from the loop. The helix E in EF1 is E1, the helix F in EF1 is F1, the helix E in EF2 is E2, the helix F in EF2 is F2, and so on. We have developed a method to analyze the conformational state of the lobe of calmodulin. In this method^6^, the EF-lobe is placed in a coordinate system by aligning its pseudo two-fold axis with z-axis of the coordinate system. Then, the interface between two EF-hands is placed on yz-plane. This coordinate system is intrinsic to each EF-lobe. Supplemental Fig. 1 shows the plot of the representative structures for holo-form (1X02), apo-form with target (2L53) and apo-form without target (1DMO).

**Figure 1:**
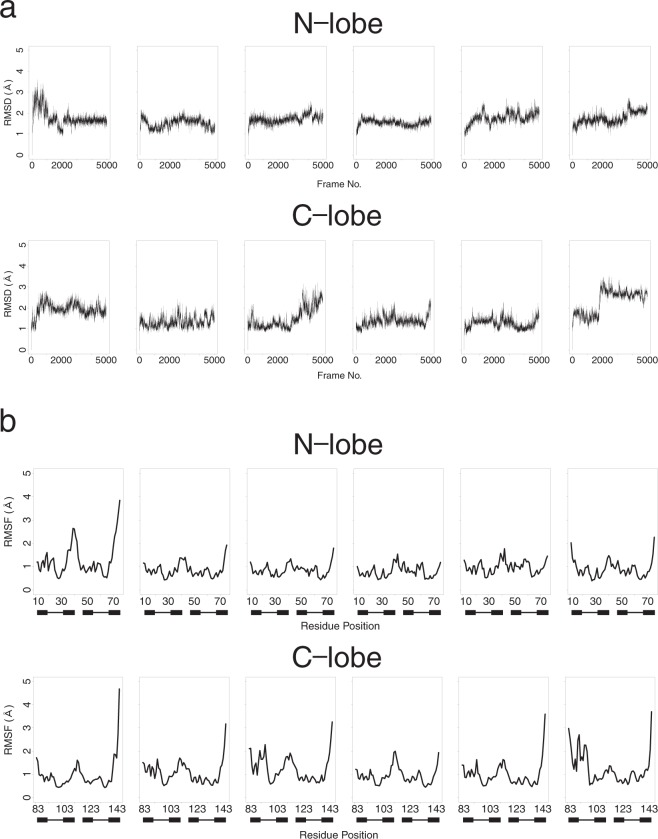
Summary of 6 × 100 ns MD of holo-calmodulin. MD simulation was performed by using GROMACS 2016 on Cray XC50 using amber99sb-ildn force field. Simulations were performed in a cubic box with periodic boundary conditions applied, where proteins were located at 14 Å distance from box boundaries. The protein was neutralized with sodium ions. After adding solvent water with tip3p model around the protein, and some of water molecules were replaced with 0.15 M NaCl, energy minimization was carried out to reach the maximum force below 250 (kJ/mol). Equilibrating the water around the protein was performed under 100 ps NVT followed by 100 ps NPT ensembles at 300 K. MD data was collected for 100 ns in the NPT ensemble at 300 K. Electrostatic interactions were calculated using the PME algorithm. This figure shows summary of six 100 ns MD run. MD trajectory was analyzed by Bio3D package of R. (**a**) Root mean square deviation (RMSD) from initial structure at each frame of 100 ns MD. The results of six MD run were analyzed for N-lobe (upper) and C-lobe (lower), each. (**b**) Root mean squared fluctuations (RMSF) at each residue of calmodulin in 100 ns MD. The results of six MD run were analyzed for N-lobe (upper) and C-lobe (lower), each. The position of EF-hand is shown with filled box (E and F-helices) and line (loop).

Figure 1 shows the root mean square deviation (RMSD) from initial model and the root mean square fluctuation (RMSF) of each residues in six 100 ns MDs starting from holo-calmodulin (1X02). Each MD run shows different profile, however, no clear differences between N- and C-lobes.

**Figure 2:**
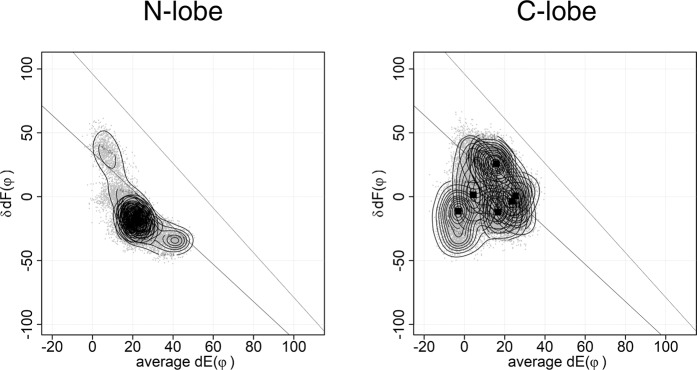
The plot of HVM of 6 × 100 ns MD. MD simulation was performed by using GROMACS 2016 as shown in Fig. 1. This figure shows HVM plot of six 100 ns MD results shown in Fig. 1. The dots are the conformations sampled at every 20 ps. The EF-lobe in the MD trajectory was placed in a coordinate system by aligning its pseudo two-fold axis to z-axis. Then, the interface between two EF-hands is placed in yz-plane. The angle between helix E and y-axis on yz-plane (dE(φ)) and the angle between two helices F in EF-lobe (δdF(φ)) are plotted. There are two lines in the plot, each of which is an inferred path of open/close of the EF-lobe based on the observed conformational continuum of EF-lobes from many EF-hand proteins. Squares show the peak position for each MD run. The contour shows the distribution of dots (EF-lobe conformation) from each trajectory of six MD runs. The distribution of EF-lobe conformation shows clear differences between N-lobe and C-lobe.

The plot of the same MD runs was made by our method (Fig. 2). There are clear differences of the plot between N- and C-lobes. The plot used here is useful to analyze the differences between EF-lobes.

**Figure 3:**
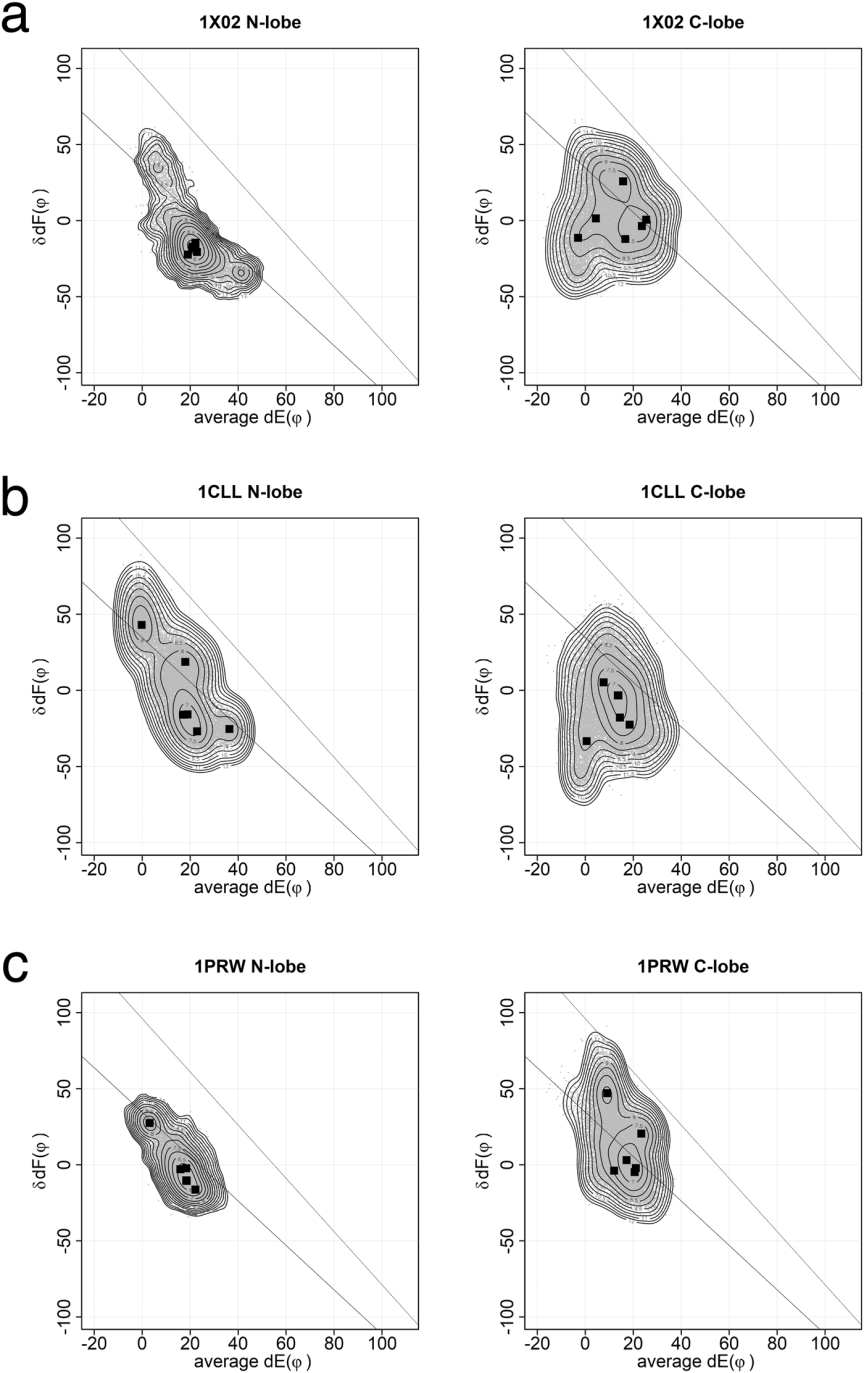
Conformational landscape of N- and C-lobes from three holo-calmodulins. This figure shows summed results of six 100 ns MD. The dots are the conformations sampled at every 20 ps. The angle between helix E and y-axis on yz-plane (dE(φ)) and the angle between two helices F in EF-lobe (δdF(φ)) are plotted. Squares show the peak position for each MD run. The contour shows the free energy landscape of ensemble summed over six MD runs. In the N-lobe, the conformations of holo-forms distribute along the line. The area of ensemble for the C-lobe is wider than that for the N-lobe. (**a**) 1X02, Xenopus holo-calmodulin (NMR). (**b**) 1CLL, Human holo-calmodulin (X-ray). (**c**) 1PRW, Bovine holo-calmodulin in a compact form (X-ray). 1PRW is holo-calmodulin in a compact form. 1CLL and 1X02 are in an extended form.

Three structures of holo-forms, two determined by x-ray crystallography (1CLL, 1PRW) and the other by NMR (1X02), were used for MD analysis. 1PRW is holo-calmodulin in a compact form. 1CLL and 1X02 are in an extended form. The plots of MD results from the three structures are similar (Fig. 3). In the N-lobe, the conformations of holo-bound forms are distributed along the lower line. Two pairs of helices E1F2 and E2F1 show coordinated movements. In the C-lobe, the conformations are not only along the line for simple open/close of pairs of helices E3F4 and E4F3, but also change the arrangement of a pair of helices, E3F4 or E4F3. This movement of the C-lobe is much more complex than N-lobe; it moves over a wider range of conformation to accommodate the target. Although N- and C-lobes share high sequence identity and superposition of holo-structures yields an rmsd of about 0.75 Å, there are significant differences in calcium affinity. Linse *et al*.^8^ reported a difference of calcium affinity between N- and C-lobes. The calcium affinity is six fold higher for the C-lobe than for the N-lobe. Martin *et al*.^9^ reported that the C-lobe shows slow dissociation and the N-lobe shows rapid dissociation of calcium. Our MD results indicate that the C-lobe, which binds calcium first, accommodates the target by adjusting its conformation. Then the N-lobe binds the target. Faga *et al*.^10^ reported that the basic residues at the linker region stabilize the N-lobe and decrease calcium affinity. Calcium affinity is affected by several factors including amino acid sequence of the calcium binding loop and stability of the EF-lobe^11^. The kinetic rate of conformational change of two EF-lobes of calmodulin in calcium binding differ more than an order of magnitude, ~20 ms for the time constant of N-lobe and ~490 μs for that of C-lobe^12^. Such kinetic differences might inherit to the structure of holo-calmodulin. The plot obtained by 6 × 100 ns MD reflects the differences of a kind of initial velocity of conformational change between N- and C-lobes. Longer MD would give the complete shape of conformational landscape of the lobes of calmodulin. The results of MD are highly dependent on the force field. We performed the same MD run using different force filed and water model (Supplemental Fig. 2). The shapes of area appeared in the plot is dependent on the force field. However, the results show also that N-lobe moves along the lower line in the plot and C-lobe moves over a wider range. This means the observed features for N- and C-lobes are not affected by the selection of force field.

**Figure 4:**
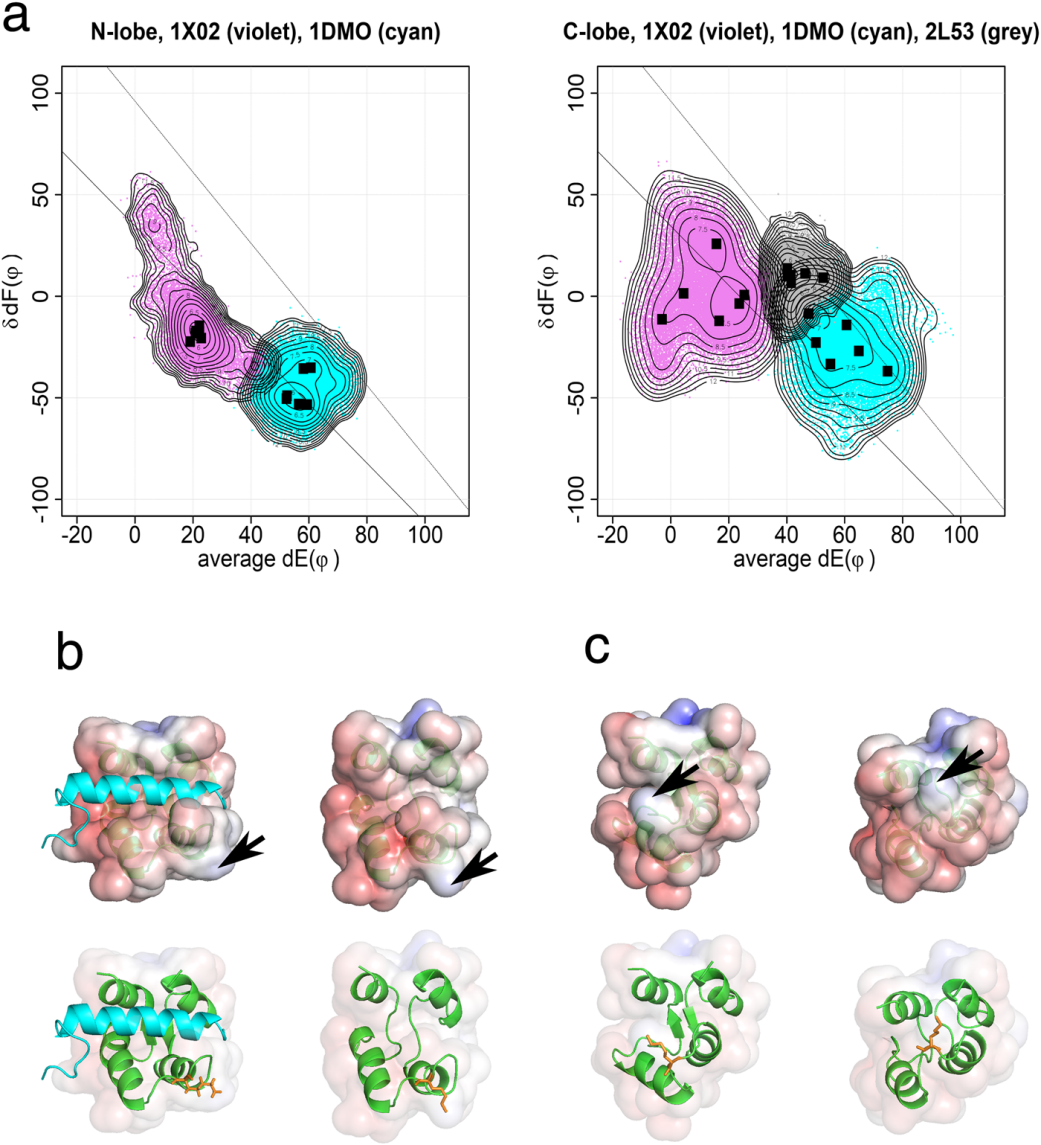
Conformational landscape of the EF-lobes from holo-, apo- and target bound apo-calmodulin. (**a**) This figure shows summed results of six 100 ns MD. The dots are the conformations sampled at every 20 ps. The angle between helix E and y-axis on yz-plane (dE(φ)) and the angle between two helices F in EF-lobe (δdF(φ)) are plotted. Squares show the peak position for each MD run. The contour shows the free energy landscape of ensemble summed over six MD runs. There are almost no overlaps of conformation between calci-forms (1X02) and apo-forms (1DMO). However, there are small overlaps between apo-forms with (2L53) and without target (1DMO). Two lines in the figure are the inferred paths for open/close of N- and C-lobes. Violet, 1X02 (calci-form); cyan, 1DMO (apo); grey, 2L53 (target bound apo-form). (**b**) Structures of C-lobe of 2L53. Left, 2L53, initial structure (model 4 of 2L53.pdb, average dE(φ) = 46.86 and δdF(φ) = 11.65) Target peptide is shown with water-blue helix. Right, sampled structure at average dE(φ) = 50 ± 0.5 and δdF(φ) = 0 ± 0.5. Target peptide is not shown. Upper, electrostatic potential. Lower, structure shown with a ribbon model; The side chain of Lys115 is shown in orange. (**c**) Structures of C-lobe of 1DMO. Left, sampled structure at average dE(φ) = 50 ± 0.5 and δdF(φ) = 0 ± 0.5. Right, 1DMO, initial structure (model 28 of 1DMO.pdb, average dE(φ) = 64.50 and δdF(φ) = −26.28). Upper, electrostatic potential. Lower, structure shown with a ribbon model; The side chain of Lys115 is shown in orange. Electrostatic potential was mapped on the solvent accessible surface. The acidic region of EF4 of C-lobe seems to pull Lys115 to prevent opening of the target binding cleft. Arrow head indicates Lys115, which is trimethylated in native calmodulin purified from mammalian sources.

We also performed MD of apo-calmodulin (Fig. 4). In the N-lobe, the conformations of holo-bound forms (1X02) and also apo-forms (1DMO) are distributed along the lower line. A two-step mechanism of calcium binding to EF-hand has been proposed based on the structural analysis of a trapped intermediate of the N-lobe of calmodulin^13^. In this model, the Ca^2+^ ion binds to the N-terminal part of the calcium binding loop initially; this generates a rigid link between helix E and the beta structure present in the center of the loop. Then helix F moves and ligates the Ca^2+^ ion with a glutamate ligand. The open/close of EF-hand is explained by the torsional flexibility of EFβ-scaffold^14,15^. There are small overlaps between holo-forms and apo-forms in the N-lobe. MD results for the N-lobe are consistent with this model. There are no overlaps between holo-forms and apo-forms in the C-lobe; these changes in the C-lobe also occur in a two-step mechanism. The range for conformational change in the C-lobe of apo-forms is wider than is that of the N-lobe. The change of the C-lobe occurs mainly along the upper line. We have speculated that the open/close of the C-lobe occurs along the upper line to the semi-open conformation of target bound apo-forms, and then goes to the position in the lower line with the helix rearrangement^7^. However, the MD results suggest that the C-lobe of apo-forms moves close to the lower line and probably the C-lobe also follows a similar conformational path of open/close to the N-lobe with this two-step mechanism. The longer MD reveals the presence of pre-formation of the calcium-bound conformation in the C-lobe of apo-calmodulin^16^; it also indicates the presence of the closed form in holo-calmodulin^17^.

The C-lobe of the apo-forms binds targets. There are small overlaps between apo-forms with (2L53) and without target (1DMO). This result indicates the interaction of calmodulin binding peptides and apo-calmodulin in the pre-formation model. We analyzed the possible path of conformational change for target binding (Fig. 4b,c). We selected the structures at the overlapped region mapped at average dE(*ϕ*) = 50 ± 0.5 and δdF(*ϕ*) = 0 ± 0.5 from 2L53 and 1DMO. The right side of Fig. 4b shows the representative structure of 2L53 at the overlapped region and the left side of Fig. 4c is the representative structure of 1DMO at the overlapped region. The structure of 1DMO at the overlapped region is close to semi-open, but the cleft for target binding blocked by a bulge of Lys115. These results probably mean that the binding of target requires some conformational change of calmodulin induced by the target and also imply the importance of the conserved Arg residue in IQ motif, which probably expels Lys115 to open the cleft. The mutation of Arg6Ala of IQ motif in neuronal voltage-gated sodium Channel Na_V_1.6 made the peptide unable to bind to calmodulin^18^. The analysis is still preliminary to conclude the model of interaction path. We need to sample all the structures comprehensively from the overlapped region.

**Figure 5:**
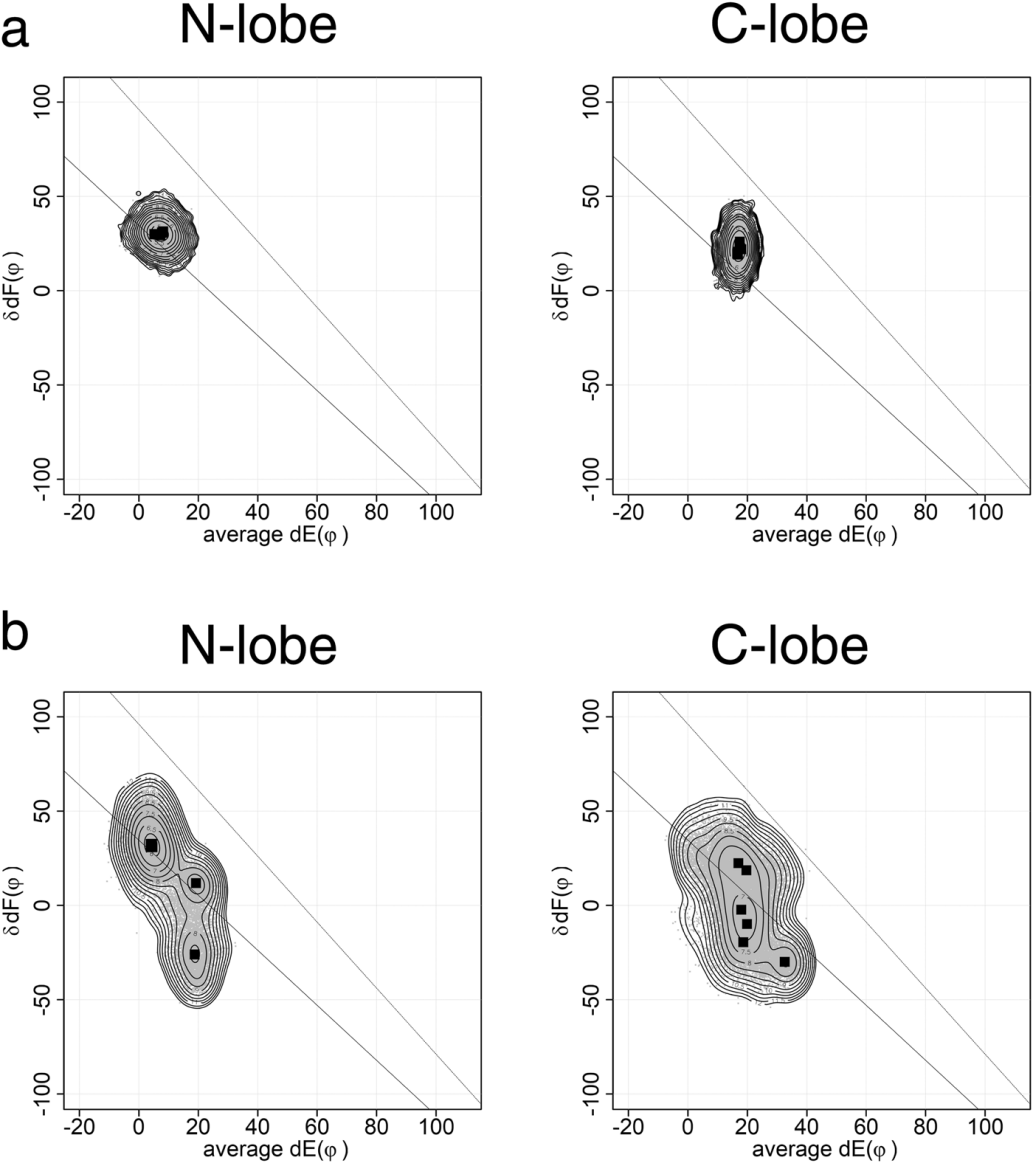
The effects of target binding for conformational ensembles of each EF-lobe. (**a**) 1IQ5, calmodulin binds calmodulin dependent protein kinase kinase (CaMKK) fragment (crystal). (**b**) MD results with a model in which the target peptide was removed from 1IQ5. The dots are the conformations sampled at every 20 ps. The EF-lobe in the MD trajectory was placed in a coordinate system by aligning its pseudo two-fold axis to z-axis. Then, the interface between two EF-hands is placed in yz-plane. The angle between helix E and y-axis on yz-plane (dE(φ)) and the angle between two helices F in EF-lobe (δdF(φ)) are plotted. Squares show the peak position for each MD run. The contour shows the free energy landscape of ensemble summed over six MD runs.

The MD results of several target bound holo-calmodulin are summarized in Supplemental Fig. 3 and Fig. 5a (1IQ5). The binding of target stabilizes both EF-lobes. The removal of the target peptide from calmodulin makes both EF-lobes more flexible (Fig. 5b). N- and C-lobes show conformational ensembles similar to those shown Fig. 3. The results also confirm that the differences between N- and C-lobe in MD behavior reflect the differences of structure between the two lobes in holo-forms.

**Figure 6:**
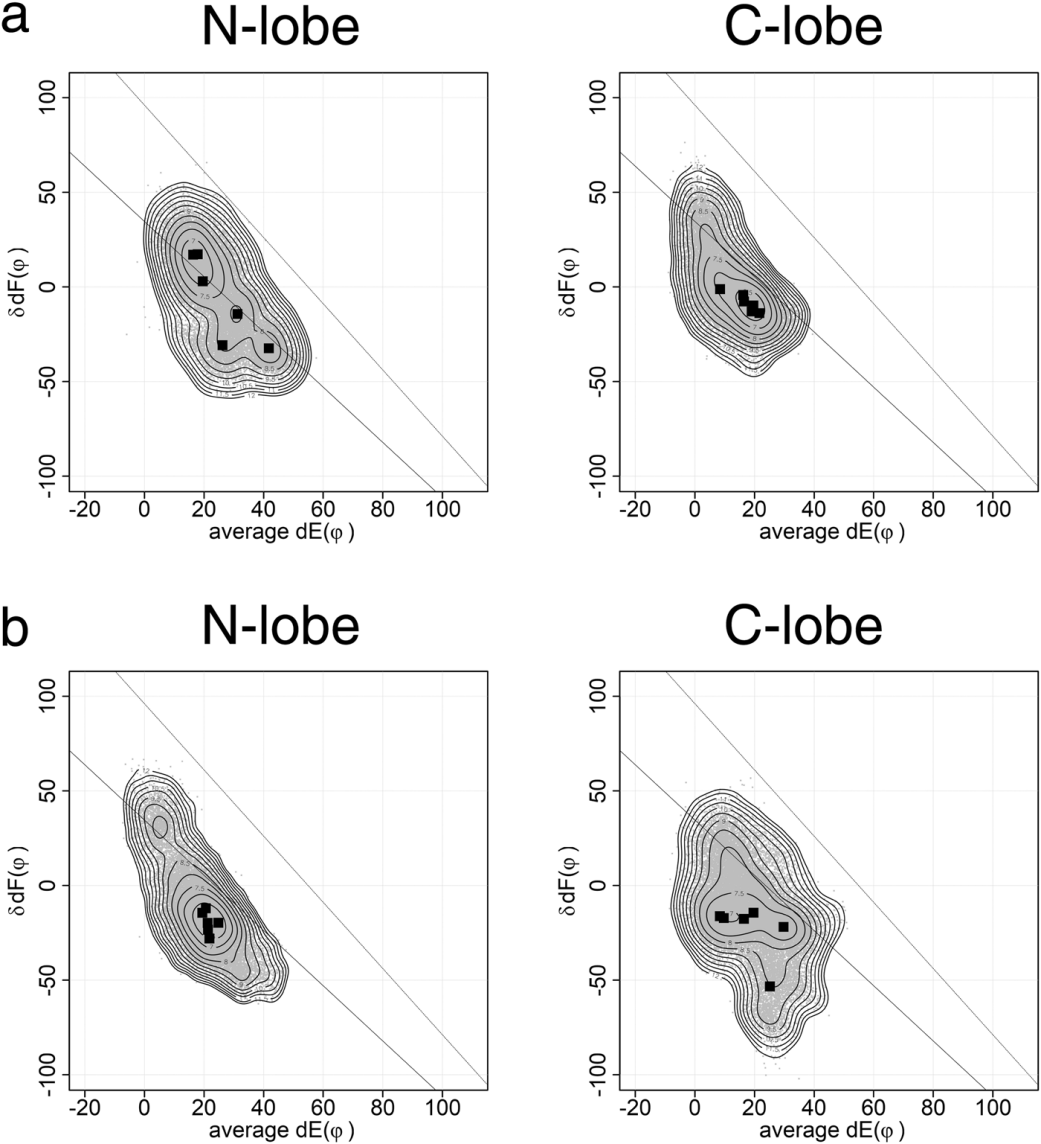
The effects of amino acid sequence for conformational ensembles of each EF-lobe. (**a**) The amino acid sequences of N- and C-lobes in 1CLL were swapped. The model was built with SwissModel by homology modelling. (**b**) The amino acid sequence of the model used in (**a**) was reverted to the native sequence. The model was built with SWISS-MODEL by homology modelling using the structure used in (**a**) as a template. MD simulations were performed by using GROMACS 2016 on a Cray XC50. This figure summarizes six 100 ns MD results for each model. The dots are the conformations sampled at every 20 ps. Squares show the peak position for each MD run. The contour shows the free energy landscape of ensemble summed over six MD runs. The models generated by SWISS-MODEL are licensed under the CC BY-SA 4.0 Creative Commons Attribution-ShareAlike 4.0 International License.

Since two lobes of calmodulin are structurally independent, the amino acid sequence, not the mutual position of lobes, should determine the differences between two lobes of holo-calmodulin observed in MD. We analyzed the effect of amino acid sequence on the difference of conformational dynamics between two EF-lobes (Fig. 6). The amino acid sequences of the N-lobe and of the C-lobe were exchanged by homology modeling; while, the sequences of the N-terminal extension and of the linker between two lobes were held constant. The MD result of this structure is shown in Fig. 6a. The C-lobe with N-lobe sequence shows the conformational change along the lower line and the major peak at a position similar to the N-lobe in Fig. 3. The N-lobe with C-lobe sequence moves over a wider area than does the native N-lobe. The change of amino acid sequences reflects the properties of their own lobe. However, these considerations do not completely explain the observed changes in conformation. This indicates additional causes for the difference between N- and C-lobes; for example, positional effects, and the interactions with the N-terminal extension and/or the linker between the two lobes. Homology-modeling with the structure used in Fig. 6a as a template and the native sequence generated another structure, which has side chains of homology-modeled conformation with the native amino acid sequence. The MD result of this structure is shown in Fig. 6b. The conformational ensemble for each lobe was returned to the one shown in Fig. 3. Since the main chain conformations of N- and C-lobes are almost identical, this result means that the differences reported here are encoded in the side chain conformations of amino acids in each EF-lobe.

**Figure 7:**
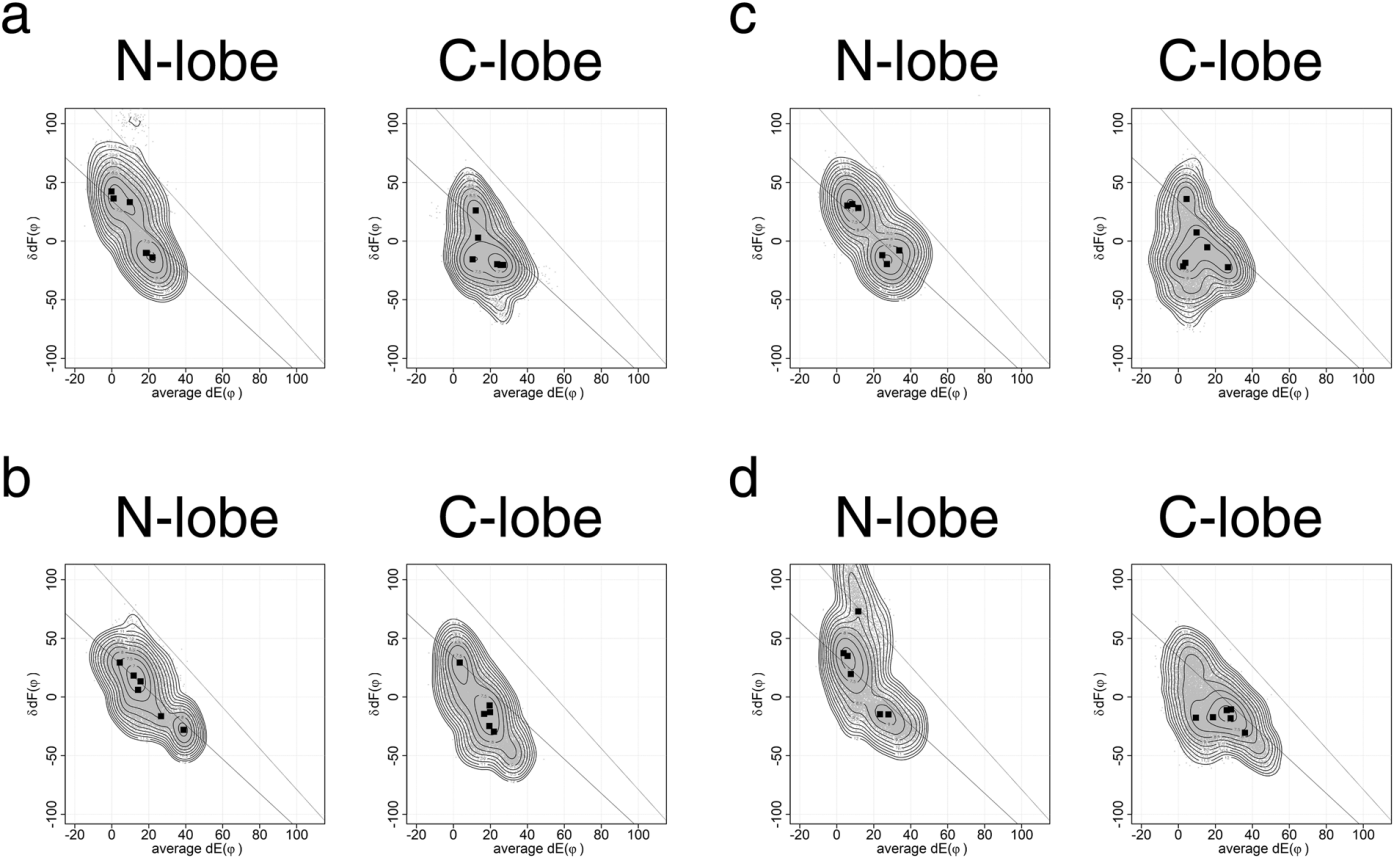
The effects of amino acid sequence for conformational ensembles of each EF-lobe. We made some mutants of calmodulin from native one and lobe-swapped one, and evaluated the effects of mutation by MD. The mutant structure was made by homology modeling with SWISS-MODEL. (**a**) F12I and L48V for N-lobe and I85F and V121L for C-lobe were mutated in 1CLL.pdb. (**b**) I12F and V48L for N-lobe and F85I and L121V for C-lobe were mutated in the lobe swapped calmodulin structure used in Fig. 6a. (**c**) F12I, I18V, T34H, P43L, L48V, V55A and P66E for N-lobe and I85F, V91I, H107T, L116P, V121L, A128V and E139P for C-lobe were mutated in 1CLL.pdb. (**d**) I12F, V18I, H34T, L43P, V48L, A55V and E66P for N-lobe and F85I, I91V, T107H, P116L, L121V, V128A and P139E for C-lobe were mutated in the lobe swapped calmodulin structure used in Fig. 6a. MD simulations were performed by using GROMACS 2016 on a Cray XC50. This figure summarizes six 100 ns MD results for each model. Squares show the peak position for each MD run. The dots are the conformations sampled at every 20 ps. Squares show the peak position for each MD run. The contour shows the free energy landscape of ensemble summed over six MD runs. The models generated by SWISS-MODEL are licensed under the CC BY-SA 4.0 Creative Commons Attribution-ShareAlike 4.0 International License.

**Figure 8:**
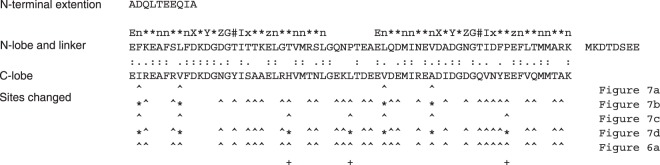
The amino acid sequences of mutant calmodulin. The mnemonic of EF-hand sequence motif and the sites changed in Figs 6a and 7a–d are shown. Asterisks for the sequence of Fig. 7b,d indicate the sites mutated from the model with swapped sequence (Fig. 6a).

By using homology modeling, we can evaluate the effects of changes of amino acid residue at each site of an EF-lobe. We evaluated the effects of amino acid sequence for conformational ensembles of each EF-lobe (Fig. 7). First we mutated the residues at the interface between two helices, helix E1 (E3) and F2 (F4) or F1 (F3) and E2 (E4). The residues at the interface were exchanged between N-lobe and C-lobe (Fig. 7a,b). The structure used in Fig. 7a is constructed by homology modeling using the native structure of 1CLL as a template. The structure used in Fig. 7b is constructed by homology modeling using a template structure whose sequence was exchanged between N- and C-lobes. Figure 7a is similar to the results of 1CLL in Fig. 3. Figure 7b is similar to Fig. 6a. The mutation only at the interface between helices did not affect the MD results (Fig. 7a,b). Then, we made mutant by adding some residues including outside of interface between helices (Fig. 7c,d). However, additional mutations affected the MD results on lobe swapped structure, especially in C-lobe (Fig. 7d), although the mutations at the same sites did not affect native structure (Fig. 7c). These three sites are important for the flexibility of C-lobe (Fig. 8). The atomic interaction of these residues against neighborhood residues might determine the differences of dynamics between N- and C-lobes. Swindells and Ikura identified two clusters conserved specifically in N-lobes or in C-lobes^19^. Shimayama and Takeda-Shitaka described the residues involved in the open-close conformational changes in EF-hand^17^. The differences of side chain conformations at conserved amino acids in each lobe might be important for the differences between two lobes. Further analyses of dynamic motion should explain the effects on atomic interactions between side chains in the EF-hand lobe.

We showed the mutant structures created by homology modeling could reproduce the difference of dynamic motion between N- and C-lobes. Our results show that the effects of mutation can be evaluated by MD using our plot. We also showed the conformational change along possible path of target binding to the C-lobe of apo-calmodulin. We used a coordinate system in the analysis of calmodulin that is based on its approximate two-fold axis. That local two-fold cannot be applied as is to the analysis of other proteins. However, the use of a local coordinate system should be applicable to other proteins. These methods can be extended to other protein families to gain greater insights into the subtle effects of amino acid replacements.

## Materials and Methods

MD simulation was performed by using GROMACS 2016^20^ on Cray XC50 using up to 4 nodes. We used amber99sb-ildn force field^21,22^. Simulations were performed in a cubic box with periodic boundary conditions applied, where proteins were located at 14 Å distance from box boundaries. The box sizes are 73–99 Å. The protein was neutralized with sodium ions. After adding solvent water with tip3p model^23,24^ around the protein, and some of water molecules were replaced with 0.15 M NaCl, energy minimization was carried out to reach the maximum force below 250 kJ/mol. Equilibrating the water around the protein was performed under 100 ps NVT followed by 100 ps NPT ensembles at 300 K. MD data was collected for 100 ns in the NPT ensemble at 300 K. Electrostatic interactions were calculated using the PME algorithm^25^. We also performed another MD using gromos53a6 force field and spc water model.

We used structures, 1DMO.pdb (Xenopus apo-calmodulin, NMR), 1CLL.pdb (Human holo-calmodulin, X-ray), 1PRW.pdb (Bovine holo-calmodulin in a compact form, X-ray), 1X02.pdb (Xenopus holo-calmodulin, NMR) 1IQ5.pdb (Xenopus target bound holo-calmodulin, X-ray) and 2L53.pdb (Human target bound apo-calmodulin, NMR), 1CKK.pdb (Xenopus target bound holo-calmodulin, NMR), 1NWD.pdb (Xenopus target bound holo-calmodulin, NMR), 2M0K.pdb (Human target bound holo-calmodulin, NMR), for the MD simulation. We selected the structure of calmodulin from Xenopus mainly. However, the structure from human was also used, when appropriate structure cannot be found in Xenopus. One structure from bovine (1PRW.dpb) was selected, since it shows a compact form in crystal. There are many structures of holo-calmodulin in PDB. However, there are quite few structures of apo-calmodulin. 1DMO and 2L53 are one of apo-calmodulins without target and with target, respectively. 2L53 is an NMR ensemble of apo-calmodulin complexed with 31-residue IQ motif peptide of voltage gated sodium channel Na_V_1.5. In this structure, the peptide interacts with the C-terminal domain (C-lobe) of calmodulin, with the N-terminal domain remaining free in solution. We have already reported the conformational mapping of NMR ensemble from 2L53.pdb^7^. We selected two models from the ensemble as the best exemplar for N-lobe and C-lobe each. 1IQ5 is a crystal structure of holo-calmodulin complexed with a 27-residue peptide of calcium/calmodulin dependent kinase kinase. Since it is a higher resolution crystal structure (1.8 Å), we used 1IQ5 as a representative for holo-calmodulin with target. We also used several structures of target bound holo-calmodulin. 1CKK is an NMR ensemble of holo-calmodulin complexed with 26-residue peptide of Ca^2+^/calmodulin dependent protein kinase. 2M0K is an NMR ensemble of holo-calmodulin complexed with 28-residue peptide of cyclic nucleotide-gated olfactory channel. 1IQ5, 1CKK and 2M0K show similar conformations with the target peptide wrapped around by N- and C-lobes of calmodulin. 1NWD is an NMR ensemble of holo-calmodulin complexed with two C-terminal peptides of glutamate decarboxylase. The N- and C-lobes of calmodulin adopt an orientation different from that seen in other calmodulin-target complexes such as 1IQ5, 1CKK and 2M0K. We performed relatively short MD several times and summed up each MD, since we focused on the conformational change of each lobe of calmodulin without the interaction between lobes.

MD results were analyzed with Bio3D^26,27^ package of R on RStudio and also analyzed by our HVM (Helix Vector Mapping) method^6,7^. MD trajectory was converted to pdb by using trjconv of gmx2016 with an option for periodic boundary conditions of cluster. This option fixes the structure broken by periodic boundary. The trajectory files of MD and the script used for the analysis will be provided upon request.

The coordinates of each EF-lobe in the trajectory was analyzed by HVM^5,6^. HVM places the EF-lobe in a coordinate system by aligning its pseudo two-fold axis to z-axis of the coordinate system. Then, the interface between two EF-hands is placed in yz-plane. This coordinate system is intrinsic to each EF-lobe. The angle between helix E and the y-axis on yz-plane (dE(φ)) and the angle between two helices F in EF-lobe (δdF(φ)) are plotted. There are two lines in the plot, each of which is an inferred path of open/close of the EF-lobe based on the observed conformational continuum of EF-lobes from many EF-hand proteins^5^. The distribution of EF-lobe conformation in the plot was estimated by two-dimensional binned kernel estimate method with bkde2D package of R. The estimated distribution was converted to free energy landscape by minus log-scaling. The contour was generated by contour function of R.

Homology modelling of calmodulin for lobe swapped mutants was performed by SWISS-MODEL^28^ with default setting of User Template option (https://swissmodel.expasy.org/interactive#structure).

Pymol^29^ and APBS plug-in^30,31^ were used to display electrostatic potential. External PQR files were used. PQR files were created by using the PDB2PQR^32^ server (http://nbcr-222.ucsd.edu/pdb2pqr_2.0.0/) with default settings but AMBER force field.

## Supplementary information

Supplemetal Figures

## References

[1] Clapham DE (2007). Calcium signaling. Cell.

[2] Hoeflich KP, Ikura M (2002). Calmodulin in Action: Diversity in Target Recognition and Activation Mechanisms. Cell.

[3] Babu YS (1985). Three-dimensional structure of calmodulin. Nature.

[4] Kretsinger RH, Rudnick SE, Weissman LJ (1986). Crystal structure of calmodulin. J. Inorg. Biochem..

[5] Kawasaki H, Kretsinger RH (2014). Structural differences among subfamilies of EF-hand proteins — A view from the pseudo two-fold symmetry axis. Proteins Struct. Funct. Bioinforma..

[6] Kawasaki H, Kretsinger RH (2015). HVM: A web-based tool for alignment of EF-hand lobes relative to their local pseudo two-fold axes. Protein Pept. Lett..

[7] Kawasaki H, Kretsinger RH (2017). Conformational landscape mapping the difference between N-lobes and C-lobes of calmodulin. J. Inorg. Biochem..

[8] Linse S, Helmersson A, Forsén S (1991). Calcium binding to calmodulin and its globular domains. J. Biol. Chem..

[9] Martin SR, Andersson Teleman A, Bayley PM, Drakenberg T, Forsén S (1985). Kinetics of calcium dissociation from calmodulin and its tryptic fragments. Eur. J. Biochem..

[10] Faga LA, Sorensen BR, VanScyoc WS, Shea MA (2003). Basic interdomain boundary residues in calmodulin decrease calcium affinity of sites I and II by stabilizing helix–helix interactions. Proteins Struct. Funct. Bioinforma..

[11] Gifford JL, Walsh MP, Vogel HJ (2007). Structures and metal-ion-binding properties of the Ca^2+^-binding helix–loop–helix EF-hand motifs. Biochem. J..

[12] Park HY (2008). Conformational changes of calmodulin upon Ca^2+^ binding studied with a microfluidic mixer. Proc. Natl. Acad. Sci..

[13] Grabarek Z (2005). Structure of a Trapped Intermediate of Calmodulin: Calcium Regulation of EF-hand Proteins from a New Perspective. J. Mol. Biol..

[14] Grabarek Z (2006). Structural Basis for Diversity of the EF-hand Calcium-binding Proteins. J. Mol. Biol..

[15] Strynadka NCJ (1997). Structural details of a calcium-induced molecular switch: X-ray crystallographic analysis of the calcium-saturated N-terminal domain of troponin C at 1.75 Å resolution 11 Edited by D. Rees. J. Mol. Biol..

[16] Shukla, D., Peck, A. & Pande, V. S. Conformational heterogeneity of the calmodulin binding interface. *Nat*. *Commun*. **7** (2016). 10.1038/ncomms10910 PMC4822001 27040077

[17] Shimoyama H, Takeda-Shitaka M (2017). Residue–residue interactions regulating the Ca^2+^-induced EF-hand conformation changes in calmodulin. J. Biochem. (Tokyo).

[18] Chichili VPR, Xiao Y, Seetharaman J, Cummins TR, Sivaraman J (2013). Structural Basis for the Modulation of the Neuronal Voltage-Gated Sodium Channel Na_V_1.6 by Calmodulin. Sci. Rep..

[19] Swindells MB, Ikura M (1996). Pre-formation of the semi-open conformation by the apo-calmodulin C-terminal domain and implications for binding IQ-motifs. Nat. Struct. Mol. Biol..

[20] Spoel DVD (2005). GROMACS: Fast, flexible, and free. J. Comput. Chem..

[21] Lindorff-Larsen K (2010). Improved side-chain torsion potentials for the Amber ff99SB protein force field. Proteins Struct. Funct. Bioinforma..

[22] Hornak V (2006). Comparison of multiple Amber force fields and development of improved protein backbone parameters. Proteins Struct. Funct. Bioinforma..

[23] Mahoney MW, Jorgensen WL (2000). A five-site model for liquid water and the reproduction of the density anomaly by rigid, nonpolarizable potential functions. J. Chem. Phys..

[24] Jorgensen WL, Chandrasekhar J, Madura JD, Impey RW, Klein ML (1983). Comparison of simple potential functions for simulating liquid water. J. Chem. Phys..

[25] Darden T, York D, Pedersen L (1993). Particle mesh Ewald: An N · log(N) method for Ewald sums in large systems. J. Chem. Phys..

[26] Grant BJ, Rodrigues APC, ElSawy KM, McCammon JA, Caves LSD (2006). Bio3d: an R package for the comparative analysis of protein structures. Bioinformatics.

[27] Skjærven L, Yao X-Q, Scarabelli G, Grant BJ (2014). Integrating protein structural dynamics and evolutionary analysis with Bio3D. BMC Bioinformatics.

[28] Waterhouse A (2018). SWISS-MODEL: homology modelling of protein structures and complexes. Nucleic Acids Res..

[29] *The PyMOL Molecular Graphics System*. (Schrodinger, LLC).

[30] Lerner, M. G. & Carlson, H. A. *APBS plugin for PyMOL*. (University of Michigan, 2006).

[31] Baker NA, Sept D, Joseph S, Holst MJ, McCammon JA (2001). Electrostatics of nanosystems: Application to microtubules and the ribosome. Proc. Natl. Acad. Sci..

[32] Dolinsky TJ, Nielsen JE, McCammon JA, Baker NA (2004). PDB2PQR: an automated pipeline for the setup of Poisson–Boltzmann electrostatics calculations. Nucleic Acids Res..

