# Supplementary material for: Molecular Dynamics Study of the Changes in Conformation of Calmodulin with Calcium Binding and/or Target Recognition
Source: Sci Rep. 2019 Jul 23;9:10688. doi: 10.1038/s41598-019-47063-1 (PMC6650393; doi:10.1038/s41598-019-47063-1)
Supplement: Supplementary file 1 — Supplemetal Figures [file 41598_2019_47063_MOESM1_ESM.docx]

Molecular Dynamics Study of the Changes in Conformation of Calmodulin with Calcium Binding and/or Target Recognition

Hiroshi Kawasaki^1*^, Natsumi Soma^1^ and Robert H. Kretsinger^2^

^1^Yokohama City University and ^2^University of Virginia

*Correspondence to kawasaki@yokohama-cu.ac.jp


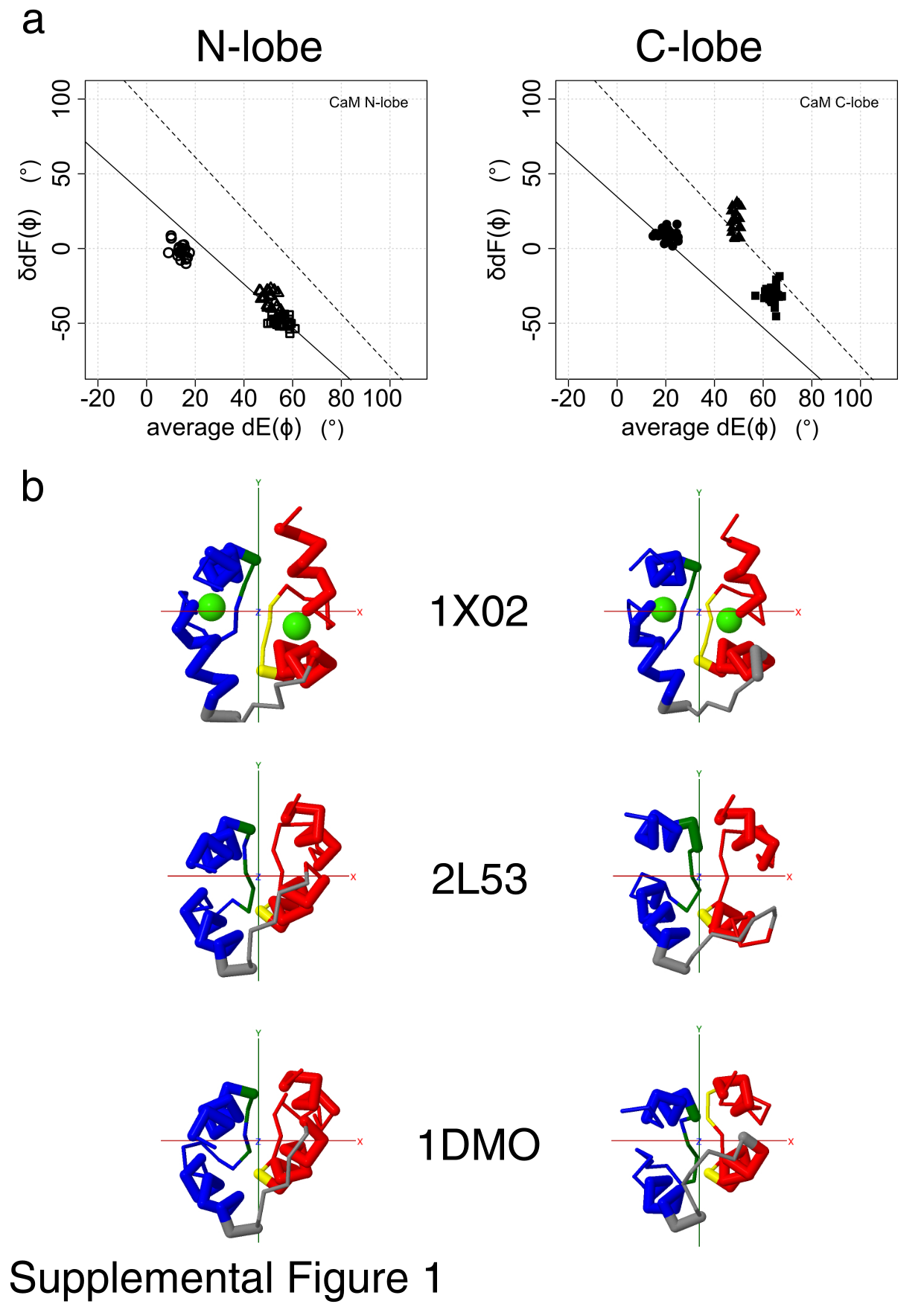


Supplemental figure 1

HVM plot and the representative structures for calcium bound-calmodulin (1X02), apo-calmodulin with target (2L53) and apo-calmodulin (1DMO)

In HVM method, the EF-lobe is placed in a coordinate system by aligning its pseudo two-fold axis with z-axis of the coordinate system. Then, the interface between two EF-hands is placed on yz-plane. This coordinate system is intrinsic to each EF-lobe. The angle between helix E and the y-axis on yz-plane (dE(φ)) and the angle between two helices F in EF-lobe (δdF(φ)) are plotted. There are two lines in the plot, each of which is an inferred path of open/close of the EF-lobe based on the observed conformational continuum of EF-lobes from many EF-hand proteins.

a)

Circle, 1X02; triangle, 2L53; square, 1DMO

b)

Structures for calcium bound-calmodulin (1X02), apo-calmodulin with target (2L53) and apo-calmodulin (1DMO)

EF-lobe is viewed down the z-axis.

Left, N-lobe; right, C-lobe

Red, EF1 for N-lobe or EF3 for C-lobe

Blue, EF2 for N-lobe or EF4 for C-lobe


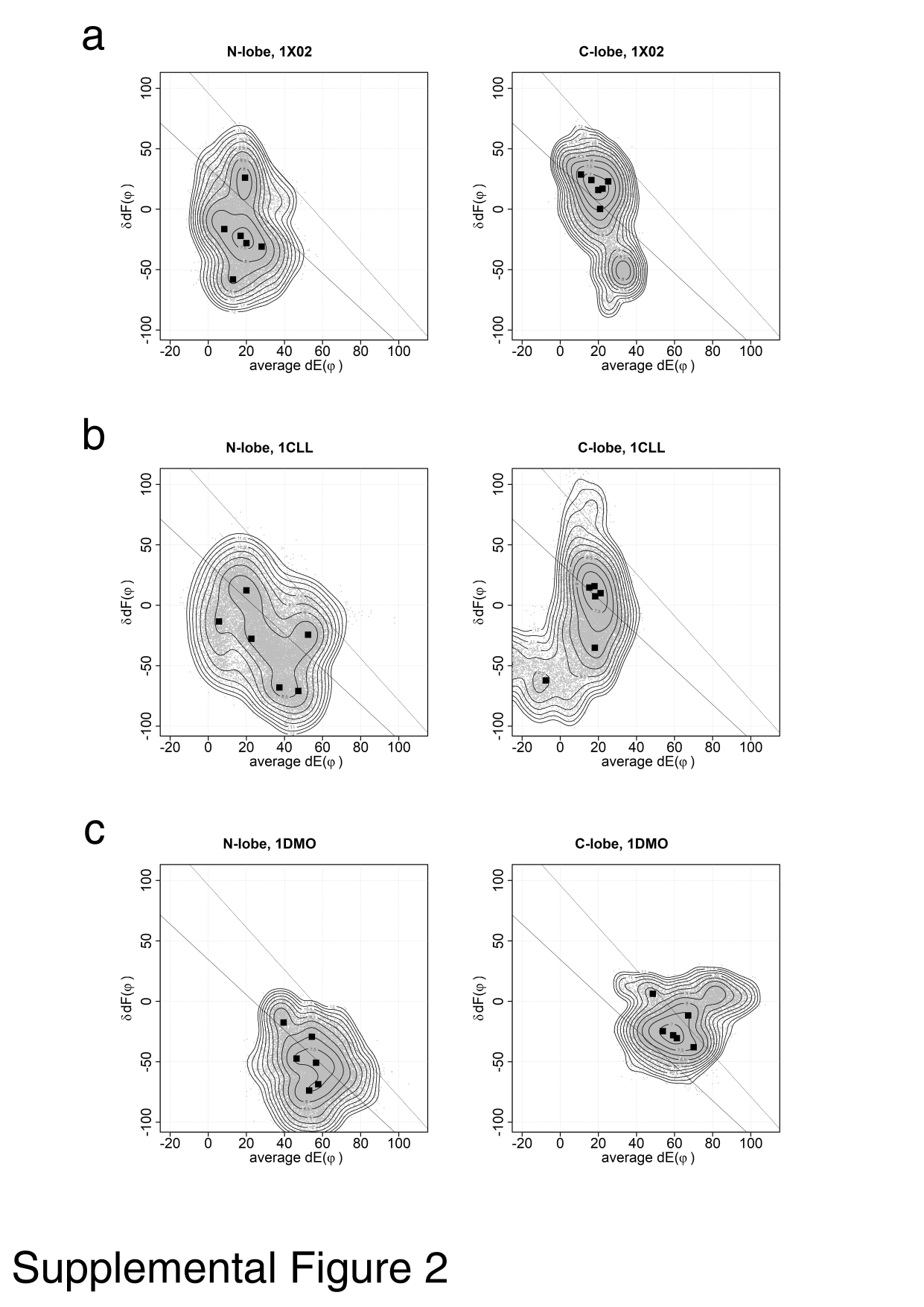


Supplemental figure 2

MD results using gromos53a6 force field and spc water model

In calcium bound-calmodulin, N-lobe moved along the line and C-lobe moved around the initial structure. In this force filed, N-lobe moved in wider area. However, the tendency of movement for N- and C-lobes is comparable with the results using amber99sb-ildn force field and tip3 water model.

1. calcium bound calmodulin (1X02)

b) calcium bound calmodulin (1CLL)

c) apo-calmodulin (1DMO)


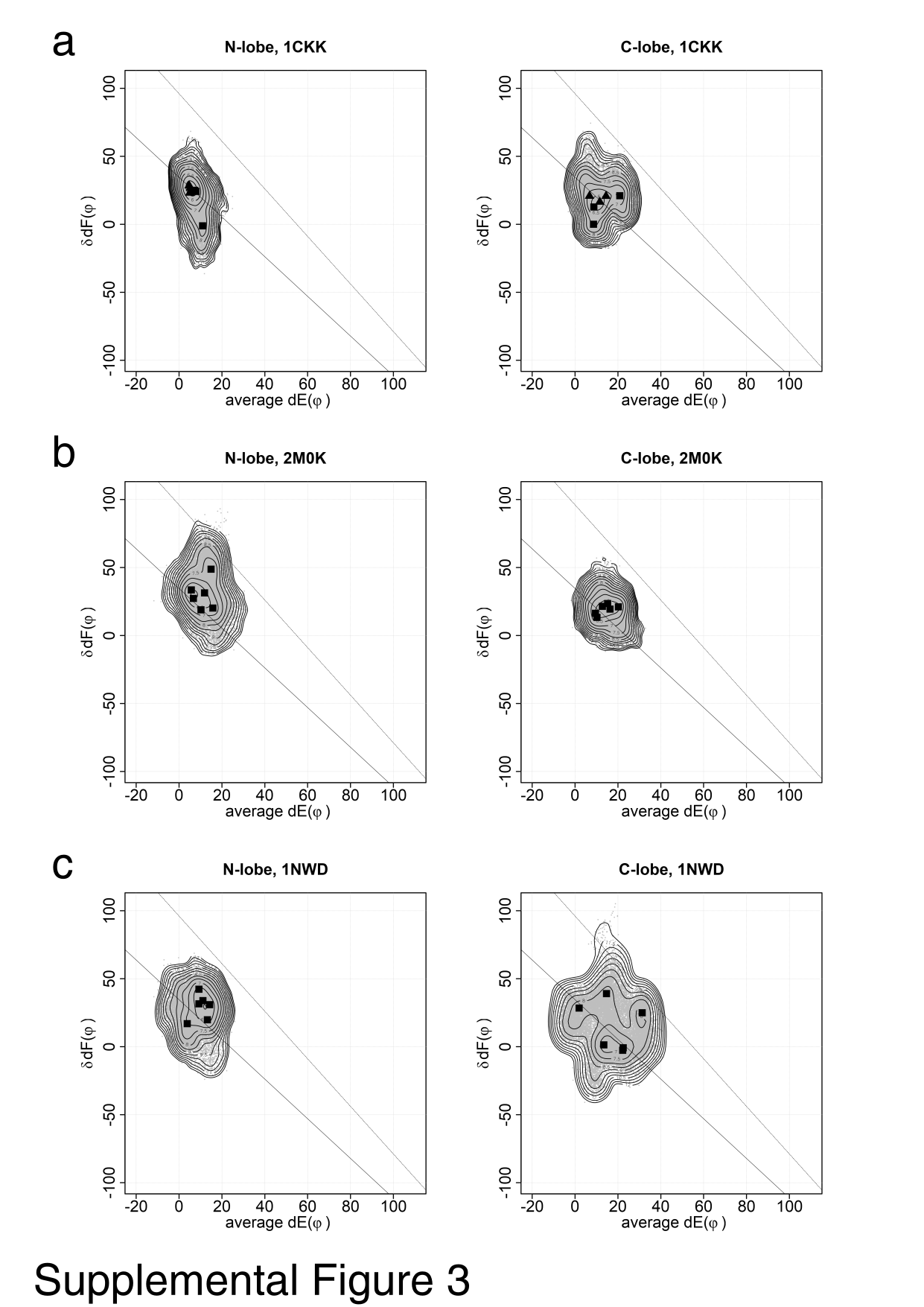


Supplemental figure 3

MD results of several target bound holo-calmodulin

1. 1CLL, Xenopus target bound holo-calmodulin; NMR ensemble of holo-calmodulin complexed with 26-residue peptide of Ca2+/calmodulin dependent protein kinase

b) 2M0K, Human target bound holo-calmodulin; NMR ensemble of holo-calmodulin complexed with 28-residue peptide of cyclic nucleotide-gated olfactory channel

c) 1NWD, Xenopus target bound holo-calmodulin; NMR ensemble of holo-calmodulin complexed with two C-terminal peptides of glutamate decarboxylase

The N- and C-lobes of calmodulin adopt an orientation different from that seen in other calmodulin-target complexes.
